# Two and three dimensional echocardiography for pre-operative assessment of mitral valve regurgitation

**DOI:** 10.1186/1476-7120-12-42

**Published:** 2014-10-25

**Authors:** Nishath Quader, Vera H Rigolin

**Affiliations:** Northwestern University Feinberg School of Medicine, Chicago, IL USA; Northwestern Medicine, 675 N St Claire, Suite 19-100, Chicago, IL 60611 USA

**Keywords:** Mitral valve, 3D echo

## Abstract

Mitral regurgitation may develop when the leaflets or any other portion of the apparatus becomes abnormal. As the repair techniques for mitral valve disease evolved, so has the need for detailed and accurate imaging of the mitral valve prior to surgery in order to better define the mechanism of valve dysfunction and the severity of regurgitation. In patients with significant mitral valve disease who require surgical intervention, multiplane transesophageal echocardiogram (TEE) is invaluable for surgical planning. However, a comprehensive TEE in a patient with complex mitral valve disease requires great experience and skill. There is evidence to suggest that 3D echocardiography can overcome some of the limitations of 2D multiplane TEE and thus is crucial in evaluation of patients undergoing mitral valve surgery. In the following sections, we review some of the crucial 2D and 3D echo images necessary for evaluation of MR based on the Carpentier classification.

## Introduction

The mitral valve apparatus is a complex structure made of the annulus, the leaflets, the chordae, the papillary muscles, and the left ventricular wall [1]. Mitral regurgitation may develop when the leaflets or any other portion of the apparatus becomes abnormal. The mitral valve apparatus may also become dysfunctional when the left ventricle dilates and the papillary muscles are displaced. As the repair techniques for mitral valve disease evolved, so has the need for detailed and accurate imaging of the mitral valve prior to surgery in order to better define the mechanism of valve dysfunction and the severity of regurgitation.

**Figure 1:**
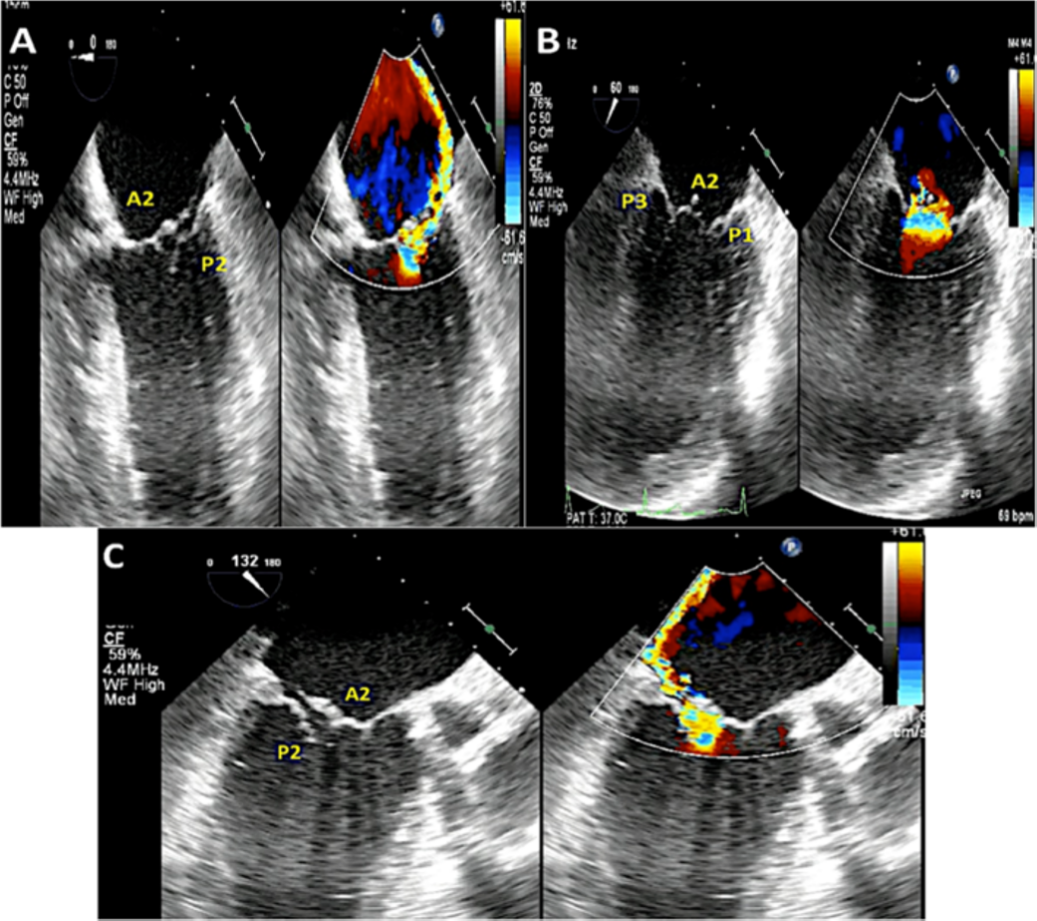
**Multiplane 2D TEE with color Doppler is utilized to identify the mitral valve scallops.** Panel **A** demonstrates a TEE at midesophageal 0 degrees view. In this view one can visualize the A2 and P2 scallops of the mitral valve at the tips of the leaflets. However the scallops visualized depend on the level of depth. At 0 degrees midesophageal view, when the aortic valve is visualized, the A1/P1 scallops are noted at the leaflet tips. When the TEE probe is advanced further into the esophagus past the midesophageal level, the A3/P3 scallops are identified at the leaflet tips [16]. Of note, the A2 scallop of the mitral valve is flail with a posteriorly directed mitral regurgitation jet. Panel **B** demonstrates a commissural view at 60 degrees. In this view, the lateral most scallop (close to the appendage) is P1. One can also visualize the central, flail A2 scallop. The P3 scallop is also well visualized in this view. The mitral regurgitation originated around the A2 scallops as was seen in the 0 degree view. Panel **C** is the long axis view between demonstrating the A2 and P2 scallops.

In patients with significant mitral valve disease who require surgical intervention, multiplane transesophageal echocardiogram (TEE) is invaluable for surgical planning. TEE can identify the mechanism of valve pathology and the specific area on the valve causing the malfunction. However, a comprehensive TEE in a patient with complex mitral valve disease requires great experience and skill [2–4], (Figure 1). Even in experienced hands, using multiplane 2D TEE alone can sometimes lead to misinterpretation of scallops. There is evidence to suggest that 3D echocardiography can overcome some of the limitations of 2D multiplane TEE and thus is crucial in evaluation of patients undergoing mitral valve surgery [5–7]. In addition, 3D TEE unifies the language used by the echocardiographer to communicate mitral valve pathology to the surgeon by providing an en face (surgeon’s view) of the mitral valve [8].

**Figure 2:**
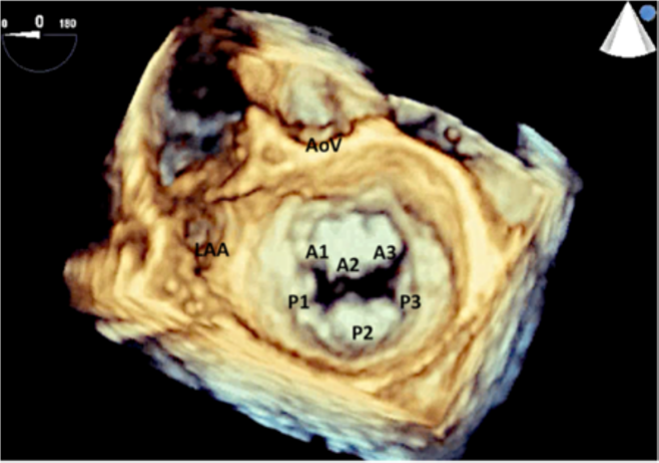
**The mitral valve has two leaflets: the anterior and posterior leaflet.** Each leaflet is further subdivided into three different scallops with the A1 and P1 scallops closest to the left atrial appendage. This view of the mitral valve is referred to as the surgeons view with the aortic valve oriented at the top of the image. LAA: left atrial appendage; AoV: aortic valve.

Mitral valve leaflet anatomy has been described by Carpentier [9] as being divided into six scallops: three that form the anterior leaflet and three that form the posterior leaflet (Figure 2). In addition, Carpentier classified the etiology of mitral valve regurgitation into Type 1-normal leaflet motion, Type II: leaflet prolapse, Type III-restricted leaflet motion. The echocardiographic must be familiar with this nomenclature as part of the preoperative assessment of mitral valve.

The following sections highlight some crucial steps that must be undertaken by the echocardiographer to accurately image the mitral valve. This includes both 2D and 3D assessment of the valve. Of note, the iE33 xMATRIX Echocardiography System (Phillips Healthcare, MA) was utilized for image acquisitions in this paper. Multiple other ultrasound companies also manufacture 3D echo systems. Image acquisition in other systems may vary slightly but the anatomic concepts are similar.

## General concepts about 3D echocardiography

The mitral valve is usually beautifully seen using 3D TEE. However, in order to obtain the best images, it is important to understand the basic concepts about image acquisition using this technology. There are three basic modes of image acquisition: Real-time 3D, 3D zoom and full volume acquisition. Each is a tradeoff between sector width, frame rate and spatial resolution.

**Figure 3:**
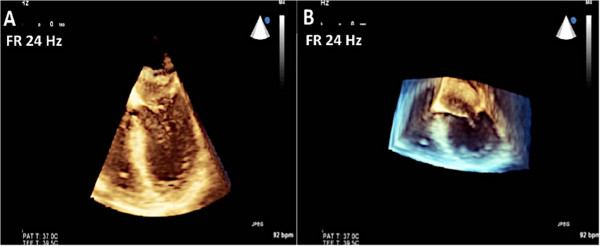
**Real time acquisition mode was used to acquire the images of the mitral valve in Panels A and B.** The high frame rate is 24 Hz. However, note the narrow sector width demonstrated in panel **B** when the data set is rotated.

**Figure 4:**
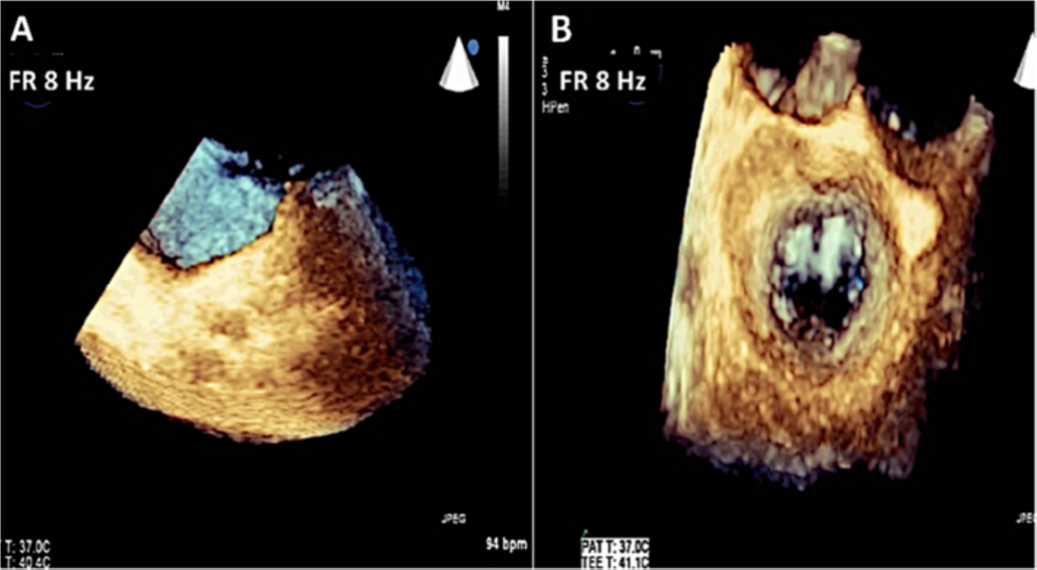
**Images of the mitral valve are acquired using the zoom mode.** The uncropped data set is seen in Panel **A**. Panel **B** shows the cropped data set demonstrating the surgeon’s view of the mitral valve. Note that the entire mitral valve is seen in this one beat acquisition but at the expense of the frame rate of 8 Hz.

**Figure 5:**
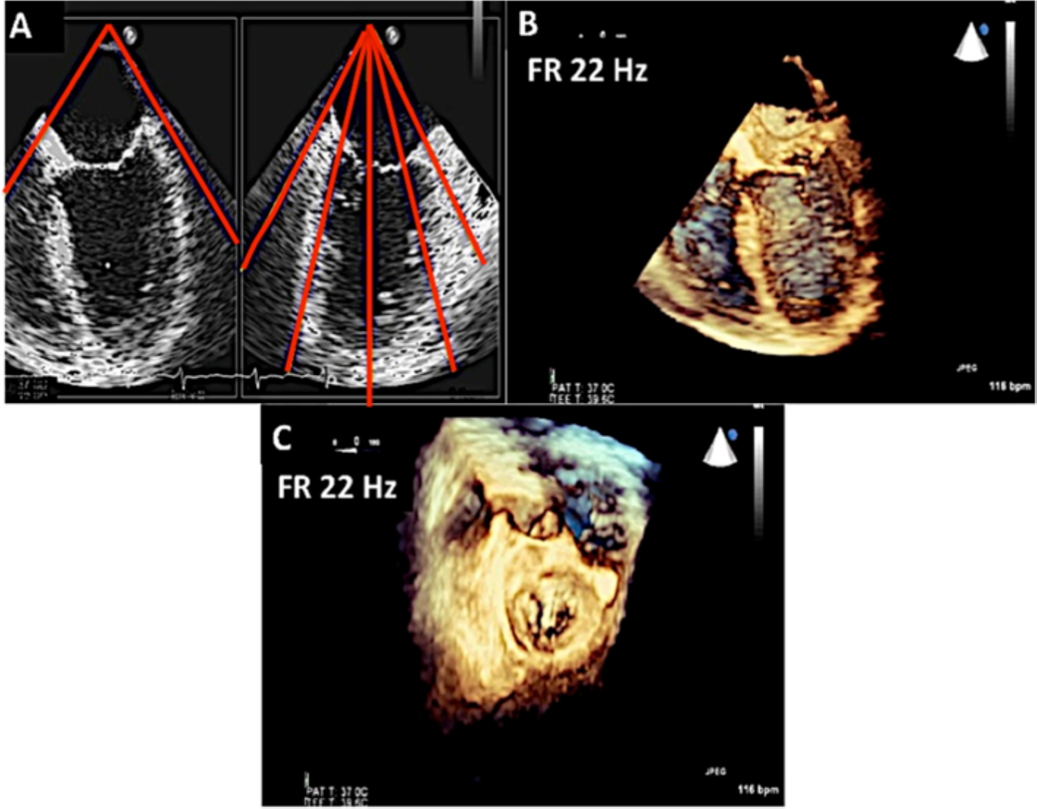
**Full volume 3D TTE: Panel A demonstrates the four volumes of data that acquired and then “stitched together to form the image in Panel B.** The data is then cropped and rotated to show the surgeon’s view of the mitral valve in Panel **C**. Note the improved spatial and temporal resolution.

**Figure 6:**
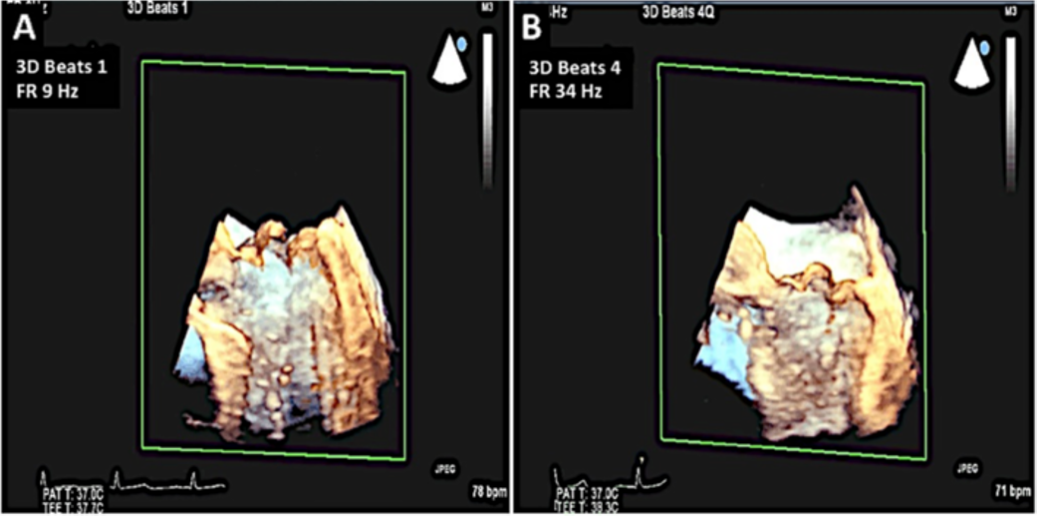
**3D echo and frame rate.** Panel **A**: 3D one beat acquisition gives a frame rate of 9 Hz. Panel **B**: A 3D four beat acquisition of the same image gives a frame rate of 34 Hz.

**Figure 7:**
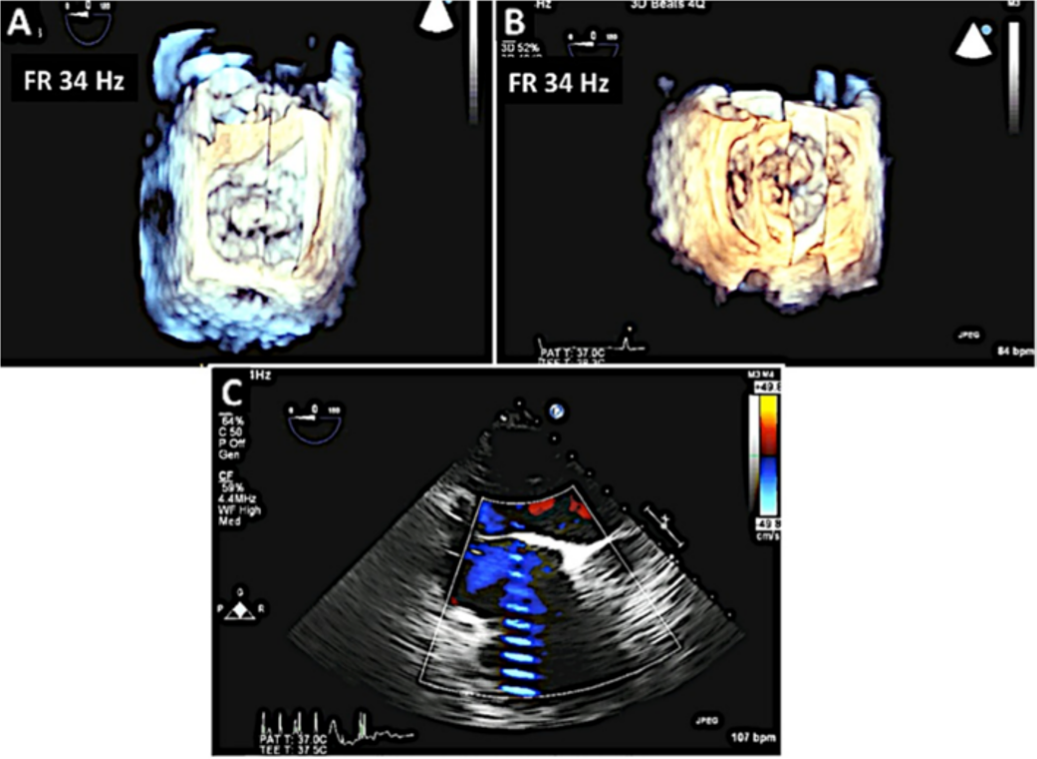
**Artifacts commonly seen on 3D and 2D TEE.** Panels **A** and **B** Significant stitch artifact due to arrhythmia. Panel **C**: The same stitch 3D artifact can also be created due to the effect of bovie on the ECG in the operating room. This bovie artifact is also present on 2D imaging.

Real-time 3D imaging provides an easy view of the mitral valve with a high frame rate but at the expense of a very narrow sector width (Figure 3). This type of imaging is useful for a quick look at small structures in a limited viewing plane. The second type of image acquisition is called the 3D zoom mode. In this case, the entire mitral valve data set can be acquired in one beat (Figure 4). This mode is useful when there are arrhythmias or a noisy ECG. The disadvantage, however, is the low frame rate and lower spatial resolution. Finally, there is the full volume acquisition mode. In this modality, multiple 3D volumes are acquired over multiple beats. The volumes are then “stitched” together in order to form the complete image. Since multiple volumes are used to create the image, both temporal (frame rate) and spatial resolution is improved (Figure 5). The echocardiographer can choose the number of beats to acquire for each full volume acquisition (usually 1–7 beats). The more beats that are used to form the image, the higher are the frame rate and image quality (Figure 6). However, this type of acquisition requires a stable ECG with a regular rhythm and no movement on the part of the patient or the echocardiographer. If the volumes cannot be properly aligned, stitch artifacts are seen in the final image (Figure 7). Color Doppler images are most often acquired using a full volume acquisition. Recently, a new mode called a “high volume rate (HVR)” mode has been developed. In this modality, the acquisition can be made in one beat with a preserved frame rate. However, the trade-off is in the spatial resolution. Thus, this mode is useful for color Doppler acquisition where frame rate and the ease of a 1 beat acquisition are the priorities. In this case, image quality is less important.

## Preoperative assessment of Type I mitral regurgitation

Carpentier classification Type I mitral regurgitation (MR) is due to a perforated leaflet or incomplete mitral leaflet closure due to annular dilation. In the case of annular dilatation, the leaflets and the subvalvular apparatus are usually normal in morphology.

**Figure 8:**
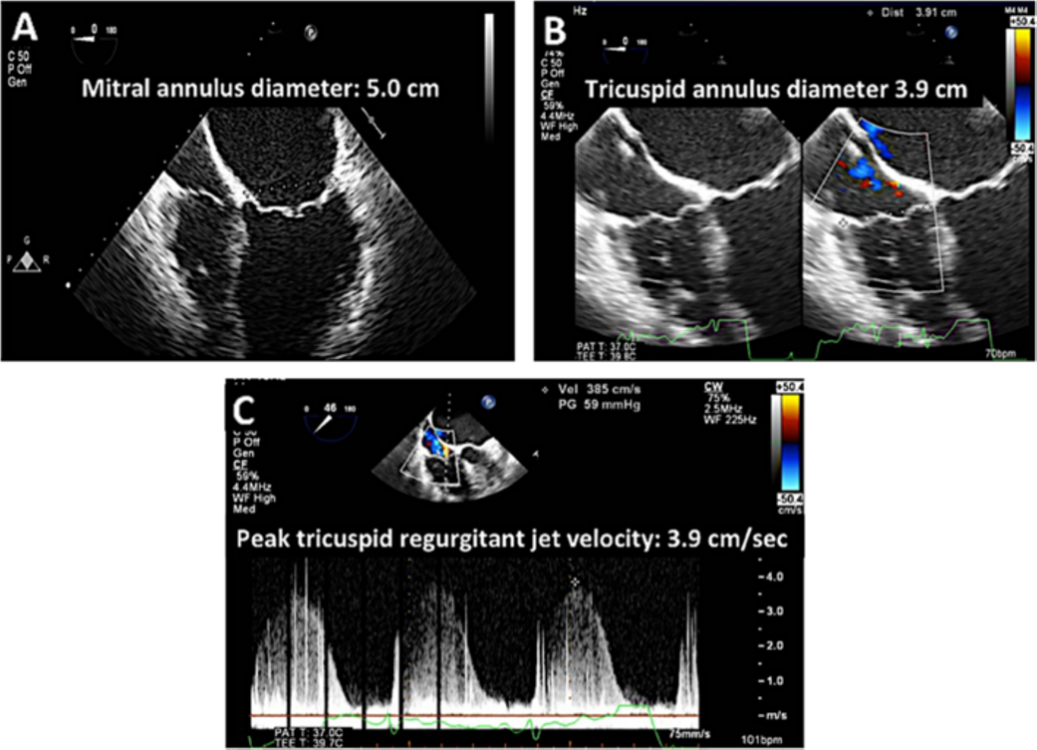
**2D assessments during preoperative TEE for MR.** Panel **A**: The mitral valve annulus is dilated (5.0 cm). Panel **B**: Tricuspid annulus should be measured as this may predict postoperative functional tricuspid regurgitation. In addition, the severity of tricuspid regurgitation should be assessed. Panel **C**: Maximum TR velocity should be measured to determine pulmonary artery systolic pressure. This is sometimes challenging on TEE and maybe better appreciated on transthoracic imaging.

The first step in evaluating for type I MR involves multiplane 2D TEE. One must recognize that one of the etiologies of the MR in this group is when the leaflets are usually normal but do not coapt as a result of annular dilatation (Figure 8). The malcoaption of the leaflets results in severe mitral regurgitation.

In addition to measuring the mitral annulus to establish that the MR is indeed due to annular dilation, the echocardiographer should also measure the tricuspid annulus (Figure 8). There have been several studies demonstrating that the pre-surgical tricuspid annulus measurement predicts residual functional tricuspid regurgitation post mitral valve surgery [10, 11]. The echocardiographer should determine the amount of tricuspid regurgitation to determine if a concomitant tricuspid annuloplasty ring is warranted at the time of mitral valve surgery. Lastly, one should also determine pulmonary artery systolic pressures since this may help in the assessment of the right ventricle post mitral valve surgery.

**Figure 9:**
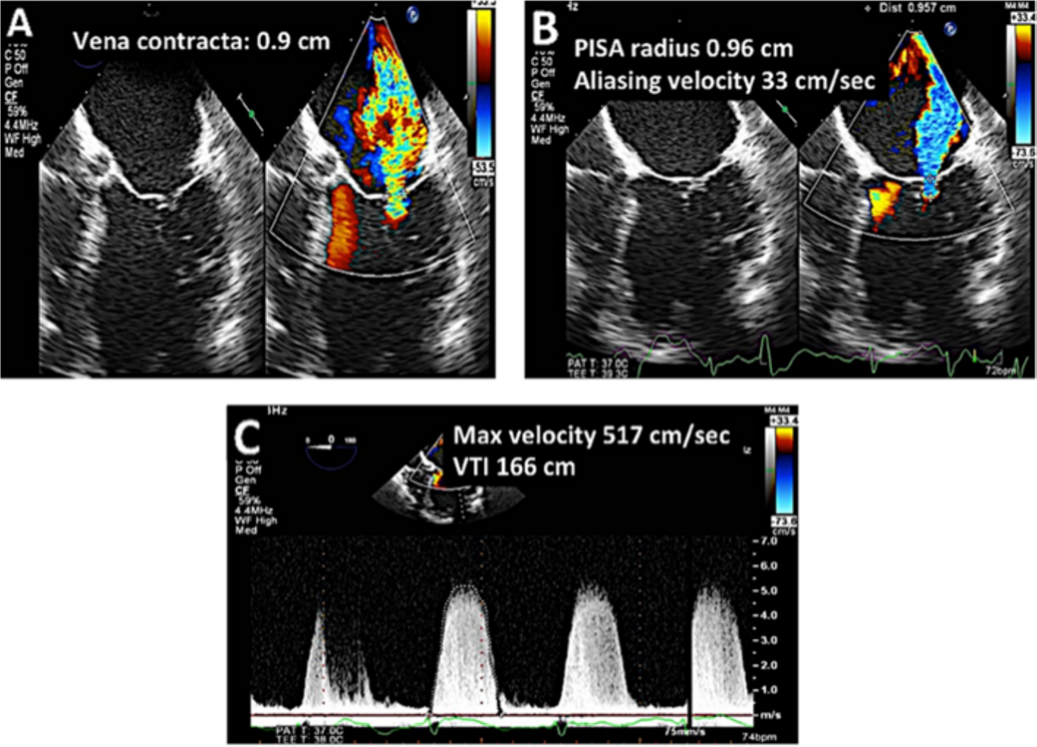
**Quantitation of MR.** Panel **A**: A vena contract >0.7 cm is consistent with severe MR. It should be measured at the narrowest portion of the MR jet. Panels **B**, **C**: Quantitation, if possible should be done by the PISA method. In this case, the EROA = 0.37 cm^2^. The regurgitant volume is 61 ml.

In addition to recognizing that there is type I MR present, the echocardiographer should also make an attempt to quantify the severity of MR according to established guidelines [12–15]. Figure 9 demonstrates the parameters needed to quantify mitral regurgitation. The echocardiographic should be familiar with the valvular heart disease guidelines and be cognizant of the qualitative and quantitative signs of severe MR [12–15].

**Figure 10:**
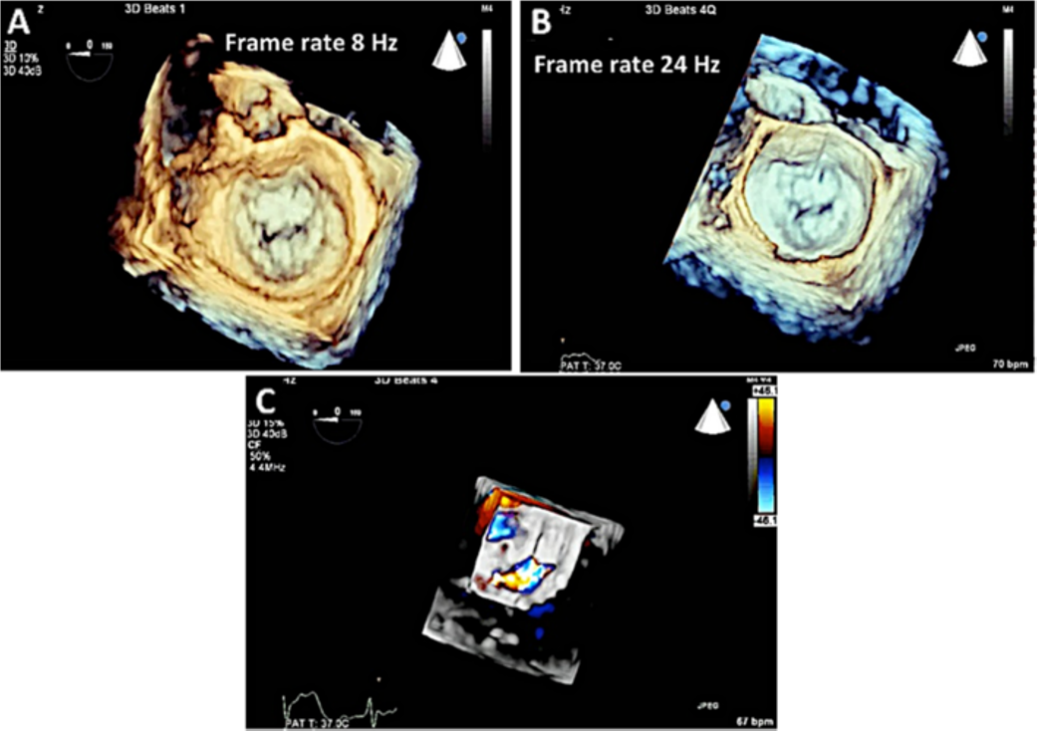
**Various 3D functions utilized to assess mitral valve disease. A**: 3D zoom function was utilized to obtain this image. The advantage of the zoom mode is that the entire data set can be acquired with one beat. However, the disadvantage is the low frame rate. In this example, the frame rate is 8 Hz. **B**: 3D full volume was used to create this image Note the frame rate of 24 Hz. **C**: Color 3D showing the origin of the jet, which is central, and throughout the coaptation line of the anterior and posterior leaflets.

**Figure 11:**
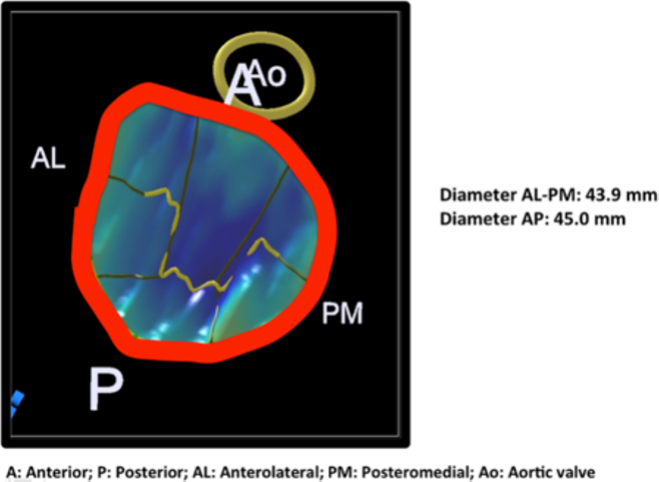
**Using the MVQ 3D function one can appreciate the shape of the mitral annulus.** In addition, a more accurate measurement of the annulus can be obtained including the anterior-posterior diameter and the anterolateral-posteromedial diameter. Figure courtesy of Nausheen Akhter, MD.

Lastly, 3D TEE is very helpful in the evaluation of type I MR. Some echocardiographers may choose to perform 3D TEE before even the 2D portion since this provides and enface view of the mitral valve and can be acquired relatively quickly. 3D TEE with color can be used to establish the origin of the MR jet (Figure 10). In addition, 3D TEE mitral valve quantification (MVQ) function can be used to assess the mitral annulus to confirm the size and shape of the annulus (Figure 11).

## Preoperative assessment of Type II mitral regurgitation

**Figure 12:**
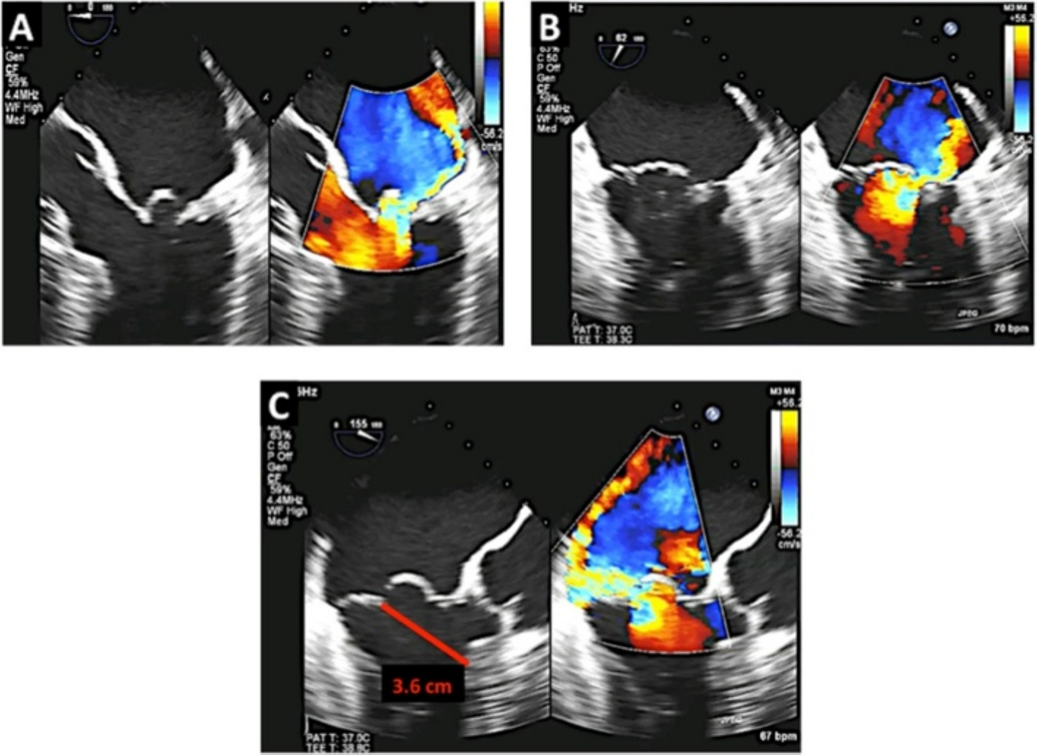
**2D and color Doppler assessment of Type II MR. A**: 2D TEE at 0 degrees demonstrates severe posteriorly directed MR due to a flail A2 scallop. **B**: TEE at the bicommisural view again demonstrates the origin of the MR. **C**: The origin of the MR is between the A2/P2 scallops. The coaptation-septum distance is also demonstrated.

**Figure 13:**
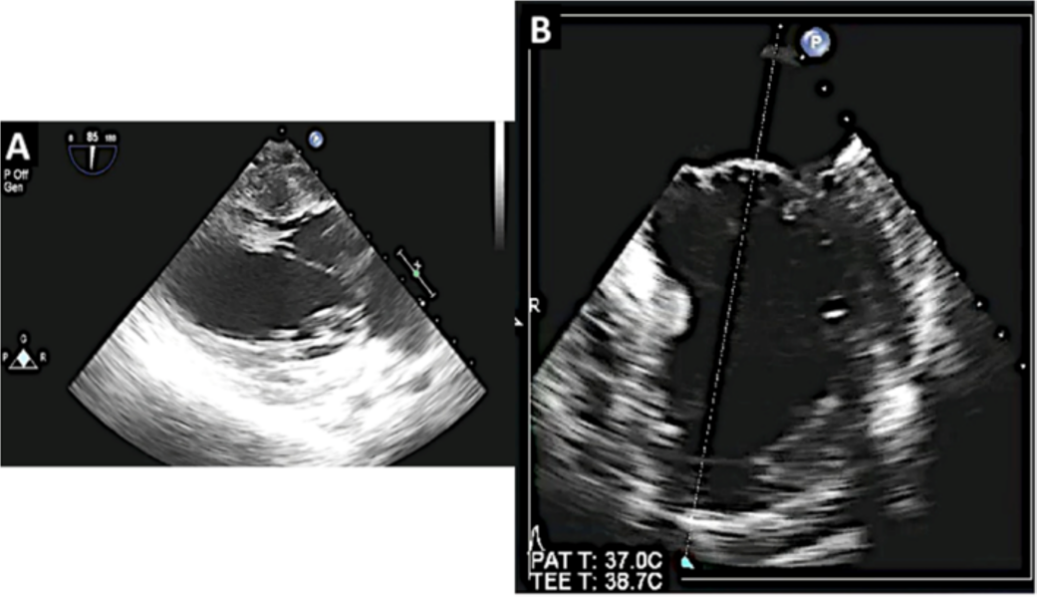
**Chamber quantification from TEE. A**: LV two chamber in a gastric view. **B**: Midesophageal two chamber view.

**Figure 14:**
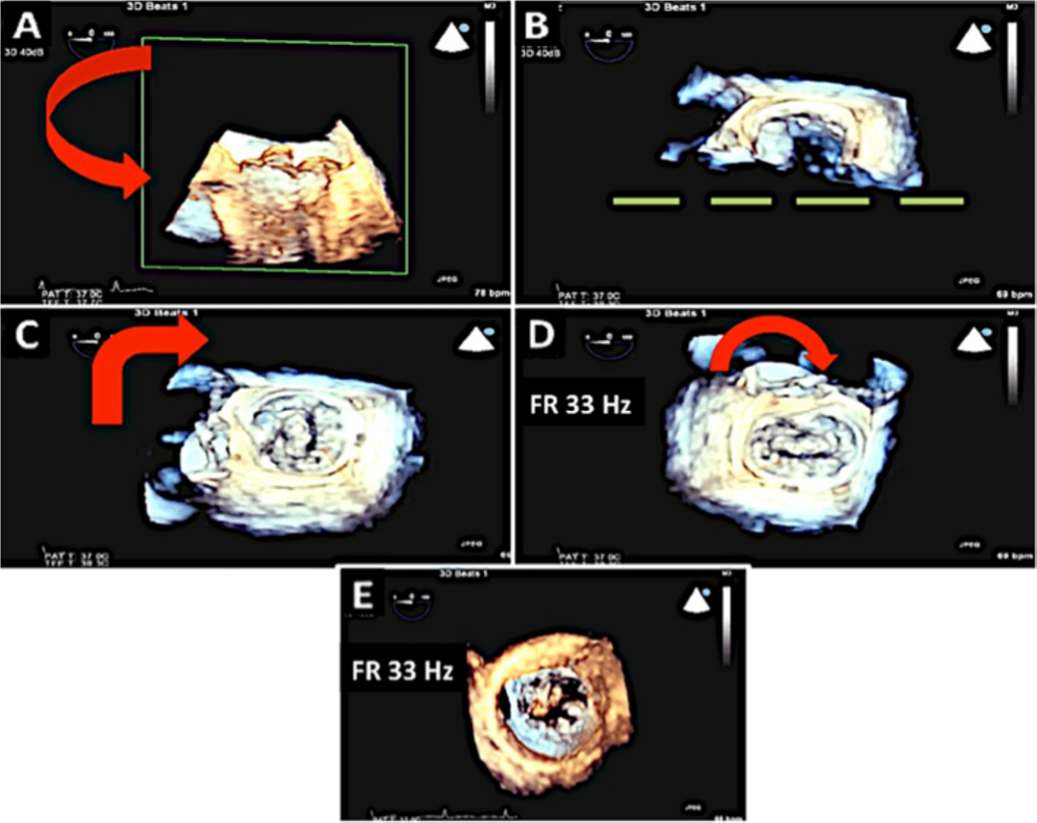
**Steps taken in 3D acquisition of mitral valve. A**: The image sector is focused on the mitral valve. Note that a one beat full volume acquisition has been performed. **B**: The image is then rotated towards the viewer. Once part of the mitral annulus is in view, the green plane is extended so that the entire mitral annulus can be viewed. **C**, **D**: The image is then rotated to position the aortic valve at the 12 o clock position. Here the mitral valve is seen from the left atrial side. **E**: The image can also be rotated so as to visualize the mitral valve from the LV side. This view can be useful to identify mitral clefts.

**Figure 15:**
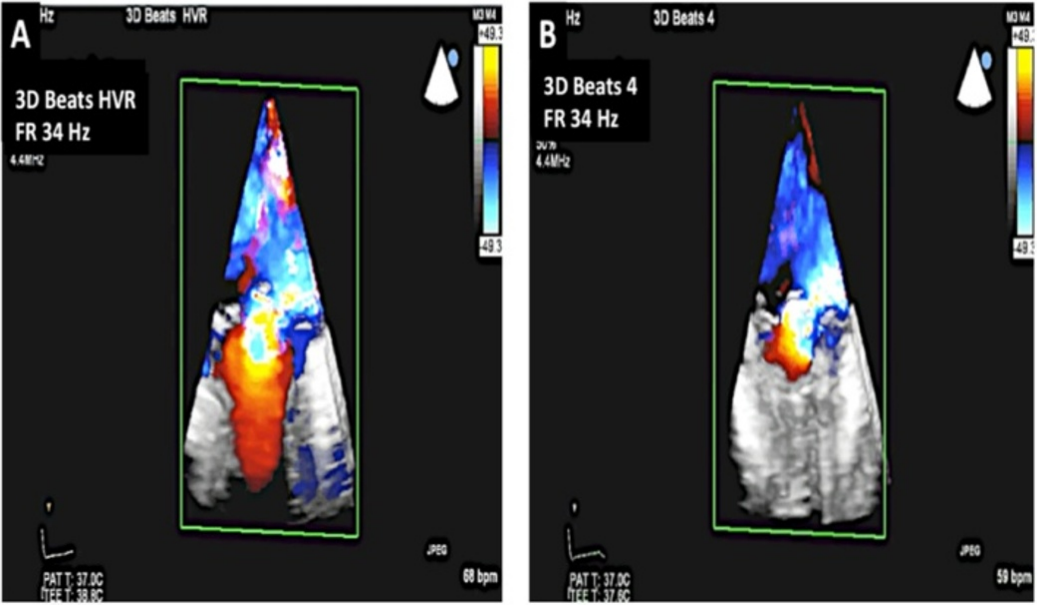
**3D color and assessment of MR. A**: In HVR mode, the location of the regurgitant jet can be identified. Also note the frame rate of 34 Hz. **B**: A 4 beat 3D acquisition can also be undertaken which gives a frame rate of 34 Hz. However, in the presence of arrhythmia, localization of the regurgitant jet may not be possible due to significant stitch artifact.

This group consists of patients with mitral valve prolapse or flail leaflet either due to Barlow’s disease or due to fibroelastic deficiency. It is important to differentiate between these two entities since this may affect surgical management. Once the pathology has been identified as mitral valve prolapse, the echocardiographer must then accurately determine the scallops involved 2D TEE can identify the diseased scallops by performing a detailed, multiplane assessment [16]. The echocardiographer should also measure the coaptation-septum distance as this is one of the determinants of postoperative systolic anterior motion of mitral valve [17]; (Figure 12). Also, the left ventricular (LV) dimensions and estimated ejection fraction should be determined. The LV chamber dimensions are measured from the midesophageal and gastric two chamber views [18]; (Figure 13).3D TEE can be acquired in a few simple steps (Figure 14). This can significantly help in the diagnosis by complementing the images obtained on 2D imaging. Once the 3D image has been acquired, a few simple post-processing steps as highlighted in Figure 14 can be performed so that the image can be oriented properly in the surgeon’s view. 3D Color Doppler imaging can then be performed to localize the origin of the regurgitant jet (Figure 15).

## Preoperative assessment of Type III mitral regurgitation

### Type 3A MR

**Figure 16:**
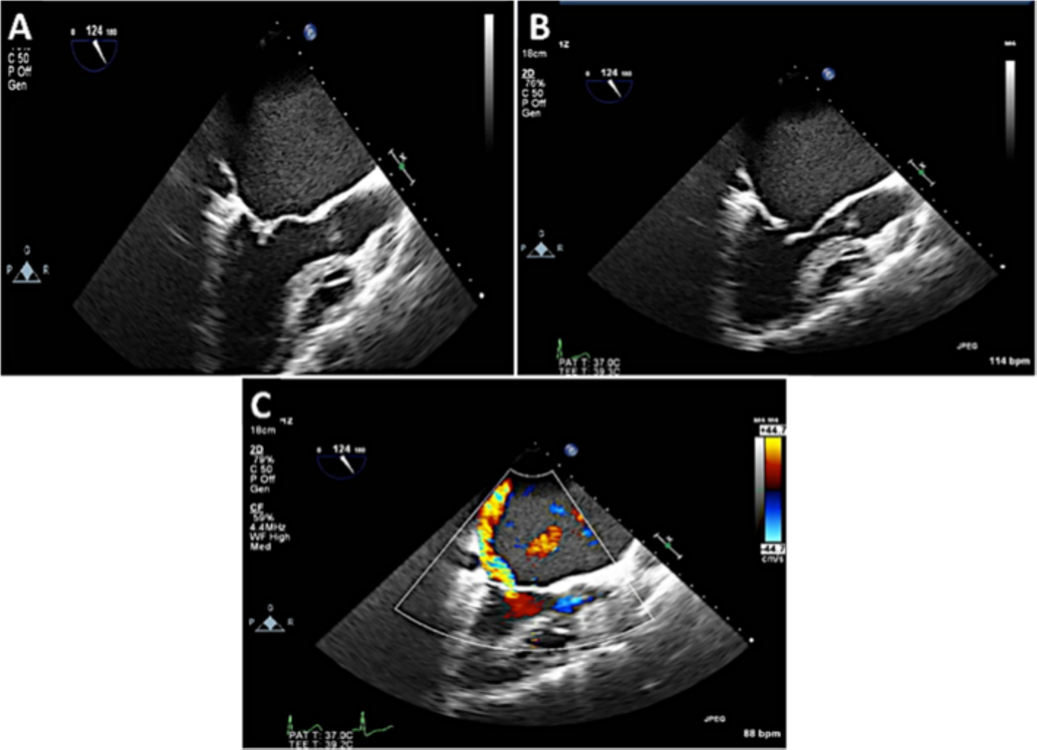
**Example of a classic rheumatic valve.** In Panel **A**, note the restricted posterior leaflet in systole. Panel **B** shows the restricted motion of the posterior leaflet in diastole as well as the hockey stick deformity of the anterior leaflet. Panel **C**: Mitral regurgitation due the restricted posterior leaflet.

**Figure 17:**
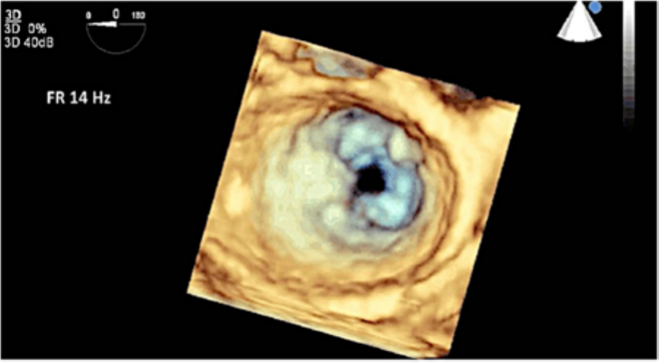
**3D full volume was utilized to image the mitral valve.** In this surgeon’s view of the valve, notice the “fish mouth” appearance of this rheumatic mitral valve due to fusion of the anterolateral commissure.

**Figure 18:**
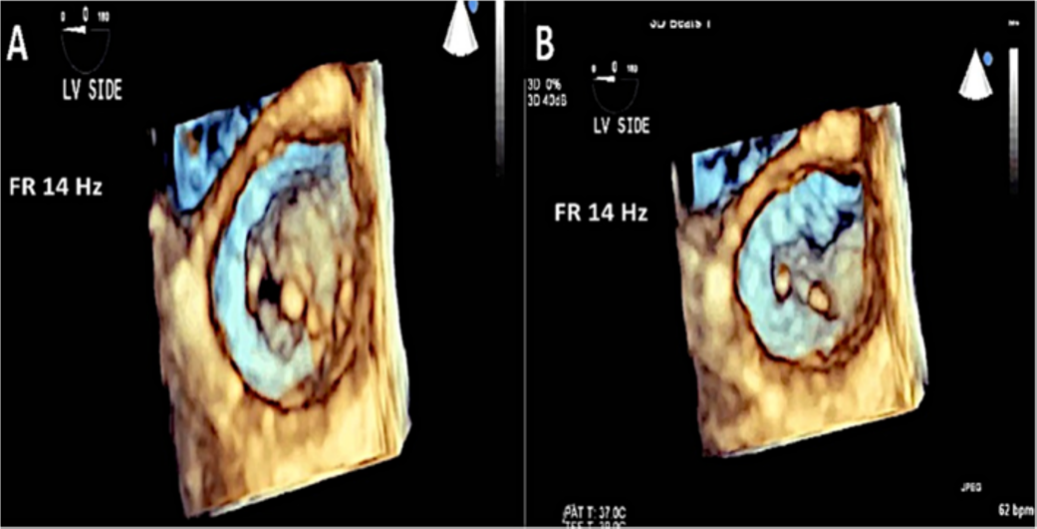
**3D TEE allows visualization of the mitral valve from the LV side confirming the diagnosis of rheumatic mitral disease. A**: Mitral valve viewed from the LV in diastole. **B**: Mitral valve viewed from the LV in systole.

This type of mitral regurgitation is caused by restricted leaflet motion in both systole and diastole. The classic example of this type of lesion is rheumatic disease. The typical rheumatic mitral valve is usually thickened. The anterior leaflet demonstrates a hockey stick deformity in diastole and the posterior leaflet is restricted in both systole and diastole (Figure 16). The restricted systolic motion of the posterior leaflet results in mitral regurgitation.3D TEE can be helpful here again to identify the classic “fish mouth” appearance of the mitral valve and to identify the extent of commissural fusion (Figure 17). One may utilize either the 3D zoom mode or a 3D full volume if the patient’s rhythm is regular. In addition, the mitral valve can be viewed from the LV side to fully appreciate the pathology (Figure 18).

### Type 3B MR

**Figure 19:**
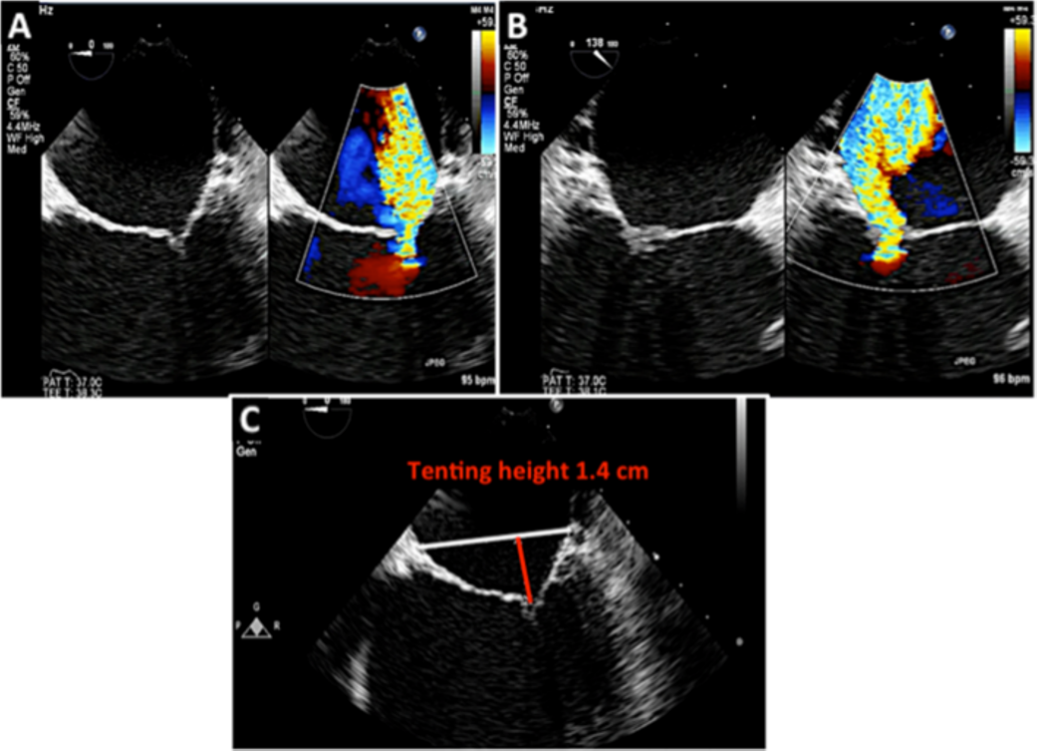
**Type 3B MR and TEE assessment. A**: Due to a right coronary artery myocardial infarction, this patient had an inferolateral wall motion abnormality with restricted motion of the posterior leaflet. This resulted in severe posteriorly directed MR. **B**: At 120 degrees the MR originates from the A2/P2 scallops. **C**: The degree of tethering should be recorded by measuring the tenting height (coaptation depth) and the area subtended between the mitral leaflets and the white line that connects the mitral annulus.

**Figure 20:**
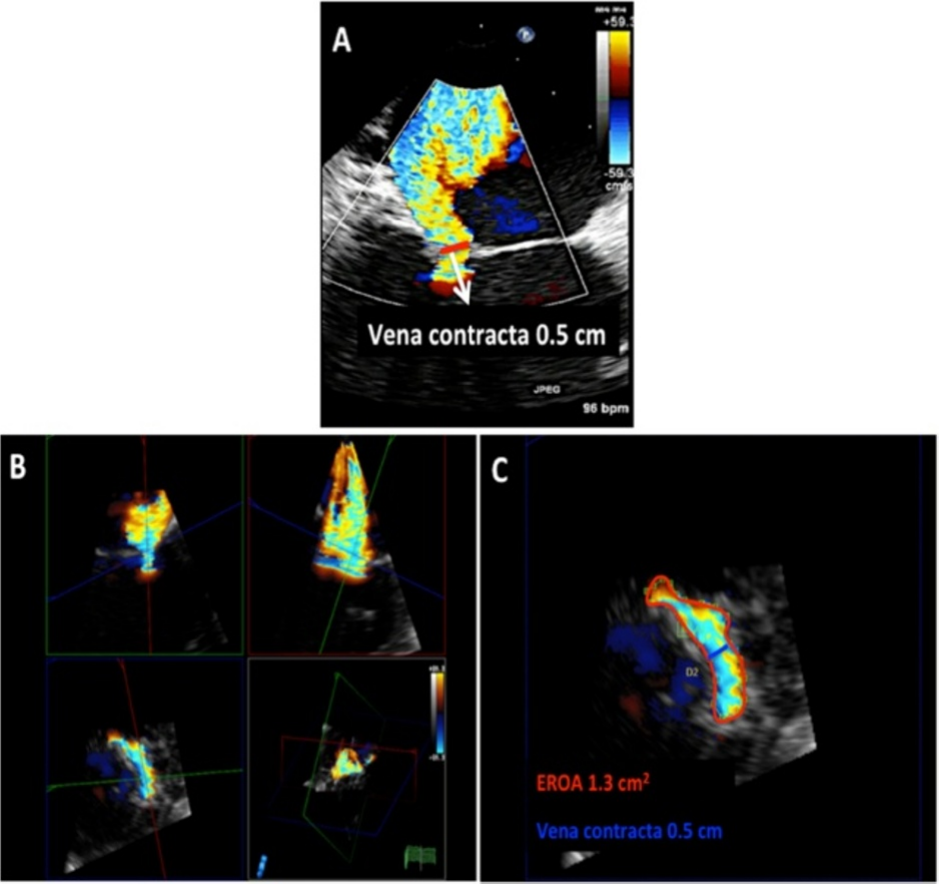
**Quantification of MR on 2D and 3D TEE. A**: The vena contracta measures 0.5 cm consistent with severe MR. **B** and **C**: MPR mode allows for aligning different planes with the regurgitant jet thus allowing measurement of the EROA en face.

This entity is classified as restricted leaflet motion in systole alone. Type 3B MR is often seen in ischemic MR where there is LV dilatation, dysfunction of the inferolateral wall and posterior papillary muscle displacement resulting in leaflet tethering and restricted motion of the posterior leaflet. Due to the abnormal coaptation of the anterior and posterior leaflets, the jet of MR is in the direction of the affected leaflet (Figure 19). In addition, the tenting height (coaptation depth) and tenting area should be measured (Figure 19, Panel C). The echocardiographer should also measure the vena contracta and the effective regurgitant orifice area (EROA). The vena contracta is measured as the narrowest portion of the jet as it regurgitates back into the left atrium [13, 14]. Figure 20 demonstrates how the vena contracta is measured. In this example the vena contracta measured 0.5 cm consistent with moderate MR. However the MR is clearly severe by visual estimation of the MR color Doppler. This is when 3D TEE and multiplanar reconstruction (MPR) can be utilized to assess the true EROA. In this 3D function, the echocardiographer can use the orthogonal planes of the regurgitant jet to obtain an en-face view of the vena contracta (Figure 20, Panel B and C) and thus can trace the EROA. Note in Figure 20C, the EROA is demonstrated along with the vena contracta. In this example the EROA was in the severe range despite what the vena contracta showed.

## Conclusion

Mitral regurgitation is a complex yet common valvular disease, which requires careful assessment to elucidate the etiology. The echocardiographer should have the knowledge and expertise to assess mitral valve disease and convey the findings to the surgeon preoperatively. 3D echo is complimentary to 2D multiple TEE and should be utilized as part of mitral valve preoperative assessment. Lastly, every attempt should be made to quantitate the amount of MR regardless of the visual impression.

## Abbreviations

2D: Two dimensional
TEE: Transesophageal echocardiogram
3D: Three dimensional
MR: Mitral regurgitation
LV: Left ventricular
MPR: Multiplanar reconstruction
MVQ: Mitral valve quantification.

## References

[1] Otto CM (2001). Evaluation and management of chronic mitral regurgitation. N Engl J Med.

[2] Foster GP, Isselbacher EM, Rose GA (1998). Accurate localization of mitral regurgitation defects using multiplane transesophageal echocardiography. Ann Thorac Surg.

[3] Enriquez-Sarano M, Freeman WK, Tribouilloy CM, Orszulak TA, Khandheria BK, Seward JB, Bailey KR, Tajik AJ (1999). Functional anatomy of mitral regurgitation: accuracy and outcome of transesophageal echocardiography. J Am Coll Cardiol.

[4] Grewal K, Malkowski M, Kramer C, Dianzumba S, Reichek N (1998). Multiplane transoesophageal echocardiographic identification of the involved scallop in patients with flail mitral valve leaflet: intraoperative correlation. J Am Soc Echocardiogr.

[5] Chauvel C, Bogino E, Clerc P, Fernandez G, Vernhet JC, Becat A, Dehant P (2000). Usefulness of three-dimensional echocardiography for the evaluation of mitral valve prolapse: an intraoperative study. J Heart Valve Dis.

[6] Hozumi T, Yoshikawa J, Yoshida K, Akasaka T, Takagi T, Yamamuro A (1997). Assessment of flail mitral leaflets by dynamic three-dimensional echocardiographic imaging. Am J Cardiol.

[7] Salustri A, Becker AE, van Herwerden L, Vletter WB, Ten Cate FJ, Roelandt JR (1996). Three-dimensional echocardiography of normal and pathologic mitral valve: a comparison with two-dimensional transoesophageal echocardiography. J Am Coll Cardiol.

[8] Tsang W, Lang RM (2013). Is 3-dimensional echocardiography essential for intraoperative assessment of mitral regurgitation?. Circulation.

[9] Carpentier AF, Lessana A, Relland JYM, Belli E, Mihaileanu S, Berebi AJ, Palsky E, Loulmet DF (1995). The physio-ring: an advanced concept in mitral valve annuloplasty. Ann Thorac Surg.

[10] Colombo T, Russo C, Cilibert GR, Lanfranconi M, Bruschi G, Agati S, Vitali E (2001). Tricuspid regurgitation secondary to mitral valve disease: tricuspid annulus function as guide to tricuspid valve repair. Cardiovasc Surg.

[11] Matsunaga A, Duran CMG (2005). Progression of tricuspid regurgitation after repaired functional ischemic mitral regurgitation. Circulation.

[12] Vahanian A, Ottavio A, Andreotti F, Antunes MJ, Baron-Esquivia G, Baumgartner H, Borger MA, Carrel TP, DeBonis M, Evangelista A, Falk V, Iung B, Lancelloti P, Pierard L, Price S, Schafers HJ, Schuler G, Stepinska J, Swedberg K, Takkenberg J, Oppell U, Windecker S, Zamorano JL, Zembala M (2012). Guidelines of the management of valvular heart disease. Eur Heart J.

[13] Nishimura RA, Otto CM, Bonow RO, Carabello BA, Erwin JP, Guyton RA, O’Gara PT, Ruiz CE, Skubas NJ, Sorajja P, Sundt T, Thomas JD (2014). 2014 AHA/ACC Guideline for the management of patients with valvular heart disease. J Am Coll Cardiol.

[14] Zoghbi WA, Enriquez-Sarano M, Foster E, Grayburn PA, Kraft CD, Levine RA, Nihoyannopoulos P, Otto CM, Quinones MA, Rakowski H, Stewart WJ, Waggoner A, Weissman NJ (2003). Recommendations for evaluation of the severity of native valvular regurgitation with two dimensional and Doppler. J Am Soc Echocardiogr.

[15] Lancelotti P, Tribouilloy C, Hagendorff A, Popesco BA, Edvardsen T, Pierard LA, Badano L, Zamorano JL (2013). Recommendations for the echocardiographic assessment of native valvular regurgitation: an executive summary from the European Association of Cardiovascular Imaging. Eur Heart J Cardiovasc Imaging.

[16] Hahn RT, Abraham T, Adams MS, Bruce CJ, Glas KE, Lang RM, Reeves ST, Shanewise JS, Siu SC, Stewart W, Picard MH (2013). Guidelines for performing a comprehensive transesophageal echocardiographic examination: recommendations from the American Society of Echocardiography and the Society of Cardiovascular Anesthesiologists. J Am Soc Echocardiogr.

[17] Varghese R, Itagaki S, Anyanwu AC, Trigo P, Fischer G, Adams DH (2014). Predicting systolic anterior motion after mitral valve reconstruction: using intraoperative transesophageal echocardiography to identify those at greatest risk. Eur J Cardiothorac Surg.

[18] Lang RM, Bierig M, Devereaux RB, Flachskampf FA, Foster E, Pellika PA, Picard MH, Roman MJ, Seward J, Shanewise JS, Solomon SD, Spencer KT, Sutton MJ, Stewart WJ (2005). Recommendations for chamber quantification: a report from the American Society of Echocardiography’s Guidelines and Standards Committee and the Chamber Quantification Writing Group, developed in conjunction with the European Association of Echocardiography, a branch of the European Society of Cardiology. J Am Soc Echocardiogr.

